# Longitudinal Lung Function Growth of Mexican Children Compared with International Studies 

**DOI:** 10.1371/journal.pone.0077403

**Published:** 2013-10-15

**Authors:** David Martínez-Briseño, Rosario Fernández-Plata, Laura Gochicoa-Rangel, Luis Torre-Bouscoulet, Rosalba Rojas-Martínez, Laura Mendoza, Cecilia García-Sancho, Rogelio Pérez-Padilla

**Affiliations:** 1 National Institute of Respiratory Diseases (INER), Mexico City, Mexico; 2 National Institute of Public Health (INSP), Mexico City, Mexico; University of Pittsburgh, United States of America

## Abstract

**Introduction:**

Our aim was to compare the longitudinal lung function growth of Mexican children and adolescents with the collated spirometric reference proposed for international use and with that of Mexican-Americans from the National Health State Examination Survey III (NHANES) III study.

**Materials and Methods:**

A cohort of Mexican children in third year of primary school was followed with spirometry twice a year through secondary school. Multilevel mixed-effects lineal models separated by gender were fit for the spirometric variables of 2,641 respiratory-healthy Mexican children expressed as Z-scores of tested reference equations. Impact of adjustment by sitting height on differences with Mexican-American children was observed in a subsample of 1,987 children.

**Results:**

At same gender, age, and height, Mexican children had increasingly higher forced expiratory volume in 1 s (FEV_1_) and Forced vital capacity (FVC) than the children from the collated reference study (mean Z-score, 0.68 for FEV_1_ and 0.51 for FVC) and than Mexican-American children (Z-score, 0.23 for FEV_1_ and 0.21 for FVC) respectively. Differences with Mexican-Americans were not reduced by adjusting by sitting height.

**Conclusions:**

For reasons that remain unclear, the gender-, age-, and height-adjusted lung function of children from Mexico City is higher than that reported by several international studies.

## Introduction

Lung function grows during infancy with acceleration during adolescence prior to attaining final lung function on average 5 years later in males than in females [1]. Lung function, an important component of the evaluation of children with respiratory problems, is influenced by gender, height, and age, but also by prenatal exposures, genetic factors, ethnicity, obesity, altitude of place of residence, tobacco smoking, air pollution, nutrition, socioeconomic level, and lung disease [2]. While for the majority of purposes having a longitudinal evaluation of pulmonary function is ideal, an individual’s lung function is usually compared with reference values obtained from cross-sectional studies, which are much more readily available [3-6]. The pattern of increase in lung function may differ if obtained from longitudinal or cross-sectional studies [7-9] because in the latter, the effects of age on lung function (lung function growth), the main objective, are confounded with secular time (period effect) and the so-called cohort effect result of the presence of the multiple birth cohorts assembled in a cross-sectional study [2]. A longitudinal study may better describe the growth spurt in adolescents [10] and the time sequence of events, such as the impact of the general state of health and nutrition, and of environmental exposures on lung growth. 

Children from Mexico City in the 8–20 years-of-age range at similar height, age, and gender had higher spirometric lung function than Mexican-American children [4]. Spirometric testing followed 1994 American Thoracic Society (ATS) [11] standards of quality in equipment and procedures. 

The present study had two principal objectives: first, to confirm whether the pulmonary function of Mexican children of the same gender, age, and height who were followed for 6 years was greater than that predicted by a study conducted in Mexican-American children from the National Health and Nutrition Examination Survey III (NHANES III) study [12] and than that predicted from recently updated reference values collated from children from several countries [13], both proposed as model reference values to be adopted in preference to those deriving from studies with small sample sizes [14]. We also wanted to see whether adjustment by sitting height reduced or eliminated lung function differences between children. At the same standing height, children who have a longer thorax, approximated by sitting height, and consequently shorter legs, would likely have larger lungs and greater lung function. 

## Materials and Methods

The Metropolitan Study to Evaluate the Chronic Effects of Pollution in School-age Children (EMPECE) was undertaken in Mexico City beginning in April 23, 1996 with children in third grade of primary school [15]. The protocol was approved by the Ethics Committee of the Mexican National Institute of Respiratory Diseases (INER). All parents provided written informed consent for the study subjects. 

Detailed methods were described [15,16], but briefly; 39 public and private primary schools were randomly selected from among all of those located within a 2-km radius of 10 automatic pollutant monitors in the Mexico City Metropolitan Area and all third-grade students were eligible. The first study phase recruited 1,819 third-grade children in an open dynamic cohort, adding new classmates in the following evaluations occurring every 6 months during the spring and autumn seasons of each year until the end of the children’s primary school education in 1999. Children remaining in the same schools studied during secondary school were followed for 3 additional years until 2002. 

Spirometry tests were conducted using identical computerized dry-rolling seal spirometers (922 Spirometer by SensorMedics, USA) that were calibrated each morning prior to data collection with a 3-L syringe (SensorMedics, USA). We recorded only the expiratory part of forced expiratory maneuvers and analyzed Forced expiratory volume in 1 s (FEV_1_), Forced vital capacity (FVC), their ratio (FEV_1_/FVC) and Peak expiratory flow (PEF). Tests were performed at the school during the morning and early afternoon hours. As many as eight forced expiratory maneuvers were conducted for each child to obtain three acceptable ones according to 1994 ATS criteria [11]. Additional details on the spirometry methodology, including a sustained quality control along the study, were described in a previous report [16].

Children with self-report of asthma, smokers, chronic respiratory symptoms (cough, wheezing, phlegm, dyspnea), or children >95% percentile of Body Mass Index (BMI) for age according to growth charts from the Centers for Disease Control and Prevention (CDC) [17] and children <8 years of age were excluded from analysis. 

The children’s FEV_1_, FVC, FEV_1_/FVC, and PEF were expressed as Z-scores (measured-predicted spirometric value for gender, age, and height/residual Standard deviation [SD], also denominated the Standard error (SE) of the estimate of the predicted value) according to three studies: first, a compilation of spirometric reference studies of children from several countries [13]; second, data from Mexican-American children from the NHANES III study [12], and third, data from a previous cross-sectional study of Mexican children [4]. A good fit to the equations would generate a mean Z-score of 0 with an SD of 1. Initial spirometric data of the children’s cohort reported in the present work were part of the cross-sectional reference equations (contributing with children 8–11 years of age) but also included older participants from high school and college up to 20 years of age [4].

We then fit multilevel mixed-effects linear models in order to determine any systematic differences between the longitudinal lung function growth of children and the three mainly cross-sectional equations, and in addition, to determine whether any difference found at time zero remained constant along the follow-up or whether longitudinal growth departed significantly from the cross-sectional models. All models included height (cm) and age (in years) as independent variables and were fit separately for boys and girls. Some ethnic differences in lung function may be attributable to varying thoracic lengths (with their proxy sitting heights) at the same standing height [18]. In multiple regression models, we compared lung function in 1,987 individuals (938 boys and 1,049 girls) from the healthy cohort, in whom sitting height was measured during the 4^th^ and 5^th^ evaluations, with 1,013 respiratory-healthy Mexican-American children (510 boys and 503 girls) from the NHANES III study of children of similar age, further adjusting by sitting height to investigate whether this reduces or eliminates spirometric function differences between them. PEF from children from Mexico City (mean altitude, 2,240 m above sea level) was compared with that of Mexican-Americans, adjusting by air density (see Section C in File S1). Additional details of the population and the statistical methods can be obtained from Online Supporting Information (see Section A and B in File S1).

The analysis was conducted using Stata ver. 11.1 program software.

## Results

Tables 1 and 2 show the participants by follow-up phase and their main characteristics. Of the 3,177 children finally included in the cohort, 536 presented at least one of the exclusion criteria as follows: 28 were <8 years of age; 67 reported having asthma; 190 were smokers, and 251 had obesity. Thus, we collected a total of 14,165 measurements from 2,641 children (1,308 boys and 1,333 girls) who were considered respiratory-healthy (Tables 1 and 2) and who were 8–17 years of age. Mean age at inclusion was 9.2 years of age (SD 1.1 years) for girls and 9.5 years (SD 1.2 years) for boys. Observations per individual ranged in number from 1‒12 observations (median, 4 observations, and interquartile range, 4 observations). The mean follow-up duration was 2.5 years (SD 1.9 years). Mean growth in height, weight, and Body mass index (BMI) compared with cross-sectional values [4] can be observed in Figure S1 in Supporting Information. On average, the cohort’s height was nearly one half of an SD below the reference values of NHANES III (SD, -0.49 ± 0.93) [17], with a lower weight (SD, -0.24 ± 0.99) and similar BMI (SD, 0.04 ± 1.1)

**Table 1:** Main characteristics of the boys studied (means and SD).

		Age	Weight	Height	BMI	FEV_1_	FVC	PEF	FEV_1_/FVC	Height-for-age	Weight-for-age
Phase	*N*	(years)	(kg)	(cm)	(kg/m^2^)	(L)	(L)	(L/s)	(%)	(Z-score)	(Z-score)
1	676	9.3 (0.7)	28.2 (4.9)	130.4 (6.1)	16.5 (1.9)	1.85 (0.31)	2.18 (0.37)	4.02 (0.87)	85.3 (7.9)	-0.73 (0.91)	-0.48 (1.19)
2	718	9.7 (0.8)	29.8 (5.3)	133.0 (6.4)	16.8 (2.0)	1.95 (0.32)	2.24 (0.37)	4.55 (0.81)	87.3 (6.4)	-0.67 (0.93)	-0.42 (1.03)
3	853	10.2 (0.8)	32.0 (6.1)	136.1 (6.6)	17.2 (2.3)	2.06 (0.33)	2.35 (0.38)	4.95 (0.87)	87.8 (5.7)	-0.56 (0.91)	-0.34 (1.07)
4	776	10.7 (0.8)	34.3 (6.8)	138.9 (7.3)	17.6 (2.3)	2.19 (0.38)	2.49 (0.42)	5.24 (0.93)	88.2 (5.6)	-0.51 (0.93)	-0.27 (1.03)
5	657	11.2 (0.8)	36.4 (7.3)	142.1 (7.7)	17.9 (2.4)	2.25 (0.41)	2.57 (0.47)	5.36 (1.01)	87.9 (5.8)	-0.42 (0.94)	-0.26 (1.02)
6	808	11.7 (0.8)	38.6 (8.1)	144.8 (8.2)	18.3 (2.5)	2.50 (0.49)	2.83 (0.54)	6.00 (1.12)	88.3 (5.7)	-0.45 (0.98)	-0.28 (1.04)
7	812	12.2 (0.8)	41.0 (8.3)	148.1 (8.2)	18.5 (2.5)	2.64 (0.54)	3.00 (0.59)	6.28 (1.23)	88.1 (6.0)	-0.34 (1.00)	-0.23 (1.02)
8	322	13.1 (0.6)	46.2 (9.0)	154.0 (8.0)	19.3 (2.6)	3.07 (0.60)	3.43 (0.64)	7.18 (1.42)	89.5 (5.6)	-0.41 (0.93)	-0.20 (1.09)
9	282	13.6 (0.6)	48.3 (9.0)	157.3 (7.9)	19.4 (2.5)	3.27 (0.65)	3.68 (0.70)	7.63 (1.54)	89.0 (5.9)	-0.44 (0.92)	-0.22 (0.97)
10	280	14.1 (0.6)	50.7 (8.9)	160.0 (7.5)	19.7 (2.6)	3.50 (0.64)	3.91 (0.70)	8.08 (1.61)	89.6 (5.9)	-0.50 (0.87)	-0.21 (0.95)
11	231	14.6 (0.5)	53.5 (8.8)	162.9 (6.9)	20.1 (2.5)	3.95 (0.69)	4.40 (0.76)	9.02 (1.74)	89.8 (6.0)	-0.54 (0.83)	-0.17 (0.92)
12	240	15.0 (0.6)	55.1 (9.2)	164.5 (6.9)	20.3 (2.8)	3.86 (0.64)	4.28 (0.72)	8.98 (1.56)	90.3 (6.3)	-0.61 (0.84)	-0.23 (0.97)

SD = Standard deviation; Phase = Study phase, evaluations twice annually during spring and autumn; BMI = Body mass index; FEV_1_ = Forced expiratory volume; FVC = Forced vital capacity; FEV_1_/FVC = ratio of FEV_1_ and FVC, PEF = Peak expiratory flow. Mean FEV_1_, FVC, and PEF in the last evaluation are slightly lower than in the 11th evaluation, but not those of all individuals evaluated were the same.

**Table 2:** Main characteristics of girls studied (means and SD).

		Age	Weight	Height	BMI	FEV_1_	FVC	PEF	FEV_1_/FVC	Height-for-age	Weight-for-age
Phase	*N*	(years)	(kg)	(cm)	(kg/m^2^)	(L)	(L)	(L/s)	(%)	(Z-score)	(Z-score)
1	776	9.1 (0.7)	28.4 (5.4)	130.1 (6.2)	16.7 (2.3)	1.72 (0.29)	1.98 (0.33)	3.81 (0.88)	87.1 (8.3)	-0.61 (0.93)	-0.42 (1.07)
2	795	9.6 (0.7)	30.0 (5.6)	132.9 (6.3)	16.9 (2.3)	1.81 (0.29)	2.04 (0.33)	4.29 (0.87)	88.9 (6.2)	-0.51 (0.89)	-0.38 (0.96)
3	949	10.1 (0.7)	32.6 (6.4)	136.6 (6.7)	17.3 (2.4)	1.96 (0.33)	2.19 (0.36)	4.80 (0.93)	89.7 (5.3)	-0.35 (0.93)	-0.30 (0.97)
4	895	10.6 (0.7)	35.2 (6.9)	139.9 (7.3)	17.9 (2.5)	2.11 (0.35)	2.33 (0.39)	5.18 (0.96)	90.5 (5.1)	-0.28 (0.96)	-0.21 (0.96)
5	748	11.1 (0.7)	37.4 (7.3)	142.7 (7.1)	18.2 (2.6)	2.18 (0.38)	2.41 (0.42)	5.34 (1.00)	90.3 (5.7)	-0.32 (0.96)	-0.20 (0.95)
6	892	11.6 (0.7)	40.2 (7.6)	145.7 (6.9)	18.9 (2.7)	2.44 (0.41)	2.69 (0.45)	6.05 (1.09)	90.8 (5.6)	-0.35 (0.93)	-0.10 (0.92)
7	912	12.1 (0.7)	42.7 (8.0)	148.7 (6.6)	19.2 (2.9)	2.57 (0.42)	2.84 (0.46)	6.36 (1.08)	90.7 (5.5)	-0.36 (0.92)	-0.05 (0.92)
8	351	13.0 (0.6)	47.8 (8.3)	152.6 (5.9)	20.5 (3.0)	2.93 (0.43)	3.19 (0.47)	7.12 (1.15)	92.1 (5.1)	-0.64 (0.86)	0.05 (0.93)
9	327	13.5 (0.6)	49.1 (7.5)	154.0 (5.6)	20.7 (2.7)	3.02 (0.43)	3.31 (0.47)	7.27 (1.18)	91.4 (5.3)	-0.73 (0.84)	0.03 (0.81)
10	317	14.0 (0.6)	50.7 (7.5)	154.9 (5.5)	21.1 (2.8)	3.09 (0.44)	3.40 (0.49)	7.40 (1.19)	91.3 (5.4)	-0.78 (0.84)	0.04 (0.79)
11	269	14.5 (0.6)	51.9 (6.9)	155.7 (5.3)	21.4 (2.5)	3.30 (0.43)	3.63 (0.48)	7.81 (1.24)	91.0 (5.7)	-0.83 (0.82)	0.03 (0.71)
12	279	14.9 (0.6)	52.0 (7.1)	155.8 (5.3)	21.4 (2.7)	3.12 (0.39)	3.43 (0.44)	7.62 (1.18)	91.4 (5.7)	-0.89 (0.83)	-0.08 (0.80)

SD = Standard deviation; Phase = Study phase, evaluations twice annually during spring and autumn; BMI = Body mass index; FEV_1_ = Forced expiratory volume in 1 sec; FVC = Forced vital capacity; FEV_1_/FVC = Ratio of FEV_1_ and FVC; PEF = Peak expiratory flow. Mean FEV_1_, FVC, and PEF in the last evaluation are slightly lower than in the 11th evaluation, but not all individuals evaluated were the same.

**Figure 1:**
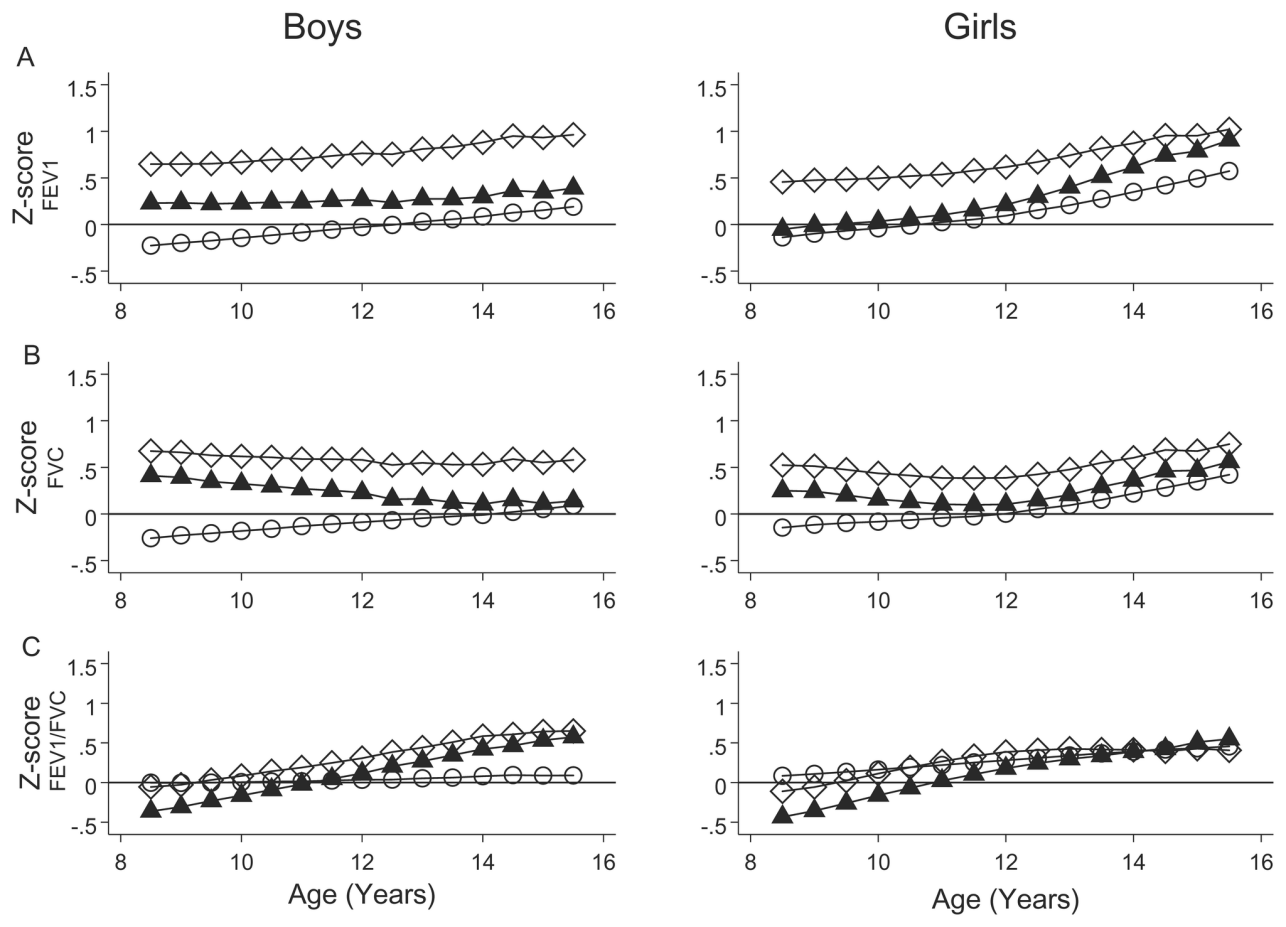
Lung function growth of a cohort of Mexican children compared with international spirometric reference equations. Forced expiratory volume in 1 s (FEV_1_) (panel A), Forced vital capacity (FVC) (panel B), the ratio of FEV_1_ and FVC (FEV_1_/FVC) (panel C) vs. age in years of children from Mexico City is expressed as Z-score of the international study of lung function by Quanjer et al. (empty diamond), Mexican-American children from the National Health and Nutrition Examination Survey III (NHANES III) study (full triangles), and Mexican children from a cross-sectional study (empty circles). The majority of values for age-, gender-, and height-adjusted FEV_1_ and FVC in Mexican children from the reported cohort are positive compared with those of children from international studies and of Mexican-American children, indicating higher spirometric lung function, and these in addition change position during growth relative to predicted values. The better the fit, the closer the median Z-score will be to zero at all ages.

Figure 1 depicts FEV_1_, FVC, PEF, and FEV_1_/FVC of the cohort expressed as Z-scores from three cross-sectional reference studies. Mexican children had a higher spirometric function than that predicted by the reference values reported by Quanjer et al. [13] Mean Z-scores (SD) for Mexican children were above zero: in boys, FEV_1_ 0.76 (1.15), FVC 0.58 (1.11), and FEV_1_/FVC 0.29 (1.04), and in girls, FEV_1_ 0.59 (1.12), FVC 0.44 (1.09), and FEV_1_/FVC 0.26 (1.05) (See Table 3 and Figure 1 [empty diamonds]). In addition, FEV_1_ and FEV_1_/FVC showed a slope vs. age that was statistically different from zero, indicating a systematic departure of longitudinal growth from the cross-sectional equations’ predicted growth (See Figure 1). Residual SD of all longitudinal models (Table 3) for FEV_1_ and FVC was about 10% higher than expected if the equation had a good fit. 

**Table 3:** Mean spirometric values of the studied population (average of all longitudinal observations) expressed as Standard deviations (SD) from three cross-sectional reference values.

Reference equation	Boys (*n* = 6,655)		Girls (*n* = 7,509)	
	Mean (SD)	*P* value	Mean (SD)	*P* value
**Quanjer et al. (13)**				
FEV_1_	0.76 (1.15)	*p* <0.001	0.59 (1.12)	<0.001
FVC	0.58 (1.11)	*p* <0.001	0.44 (1.09)	<0.001
FEV_1_/FVC	0.29 (1.04)	*p* <0.001	0.26 (1.05)	<0.001
**NHANES III (12)**				
FEV_1_	0.28 (1.02)	*p* <0.001	0.19 (1.03)	<0.001
FVC	0.25 (0.97)	*p* <0.001	0.18 (0.97)	<0.001
FEV_1_/FVC	0.09 (1.14)	0.003	0.05 (1.07)	0.048
PEF	0.77 (0.87)	*p* <0.001	0.68 (1.01)	<0.001
PEFadj	0.17 (0.77)	*p* <0.001	0.03 (0.89)	0.21
**Pérez-Padilla et al. (4)**				
FEV_1_	-0.04(1.00)	0.11	0.06 (0.96)	0.008
FVC	-0.13 (1.00)	*p* <0.001	-0.02 (0.97)	0.42
FEV_1_/FVC	0.07 (0.99)	0.005	0.26 (0.96)	<0.001
PEF	0.23 (0.90)	*p* <0.001	0.33 (0.94)	<0.001

*P* value testing the hypothesis that the measurement does not differ from zero, taking into account repeated measurements and the study sampling. Boys contributed with 6,655 observations and girls with 7,509. Ideal fit of equations would be a mean Z-score of 0 with an SD of 1. NHANES III = National Health and Nutrition Examination Survey; FEV_1_ = Forced expiratory volume at 1 sec; FVC = Forced vital capacity; FEV_1_/FVC = ratio of FEV_1_ and FVC; PEF = Peak expiratory flow; PEFadj = PEF in Mexico City adjusted for that expected at sea level, see Online Supporting Information.

Figure 1 (full triangles) and Table **3** also confirm that the gender-, age-, and height-adjusted lung function of the cohort of Mexican children was higher than that reported for the Mexican-American children from the NHANES III study during follow-up, although with mean values closer to zero and with an SD closer to 1 than values from the Quanjer et al. study. 

The study’s mean values remained above zero and increased with age for FEV_1_ in girls, while in boys these remained relatively constant (full triangles, Figure 1). For FVC, Z-scores in girls were near zero until 12 years of age, and afterward exhibited a positive slope, but in boys these demonstrated a negative slope, approaching zero at the end of follow-up (Figure 1). 

On average, FEV_1_ measured in the cohort was 170 mL higher than that predicted by Quanjer et al., 70 mL more than that of Mexican-Americans from the NHANES III study, and 30 mL more than those predicted by the Mexican cross-sectional study. For FVC, similar values were found, of 160, 70, and 10 mL, respectively, and for FEV_1_/FVC, these were 1.8, 0.4, and 0.5%, respectively (see Table S1 and Figures S3 and S4 in Online Supporting Information). The PEF Z-score (compared with Mexican-American children from the NHANES III) was 0.8 in boys and 0.7 in girls, equivalent to 0.79 L/s higher in boys and 0.65 L/s in girls than that reported in NHANES III, and remained higher after adjusting by altitude air density (see Table 3). Lung function growth of the cohort of Mexican children departed little from the cross-sectional equation reported by Pérez-Padilla et al. [4], except in girls after 12 years of age (Table 4 and Figure 1). 

**Table 4:** Multiple regression equations for spirometric variables comparing cross-sectional predicted values and longitudinal models from the reported cohort.

	Log FEV_1_ (mL)		Log FVC (mL)		FEV_1_/FVC	
Variable	Cross-sectional	Longitudinal	Cross-sectional	Longitudinal	Cross- sectional	Longitudinal
**Boys**						
Intercept	5.34	5.35	5.66	5.7	71.2	70.06
Height (cm)	0.01445	0.01326	0.0131	0.01172	0.121	0.13
Weight (kg)	0.0028	0.00387	0.0044	0.00489	-0.133	-0.09338
Age (years)	0.023	0.03248	0.0189	0.03024	0.354	0.22503
AIC*	-2,722	-27,700	-2,971	-28,300	-3,711	-42,009
SD (residual)	0.1369	0.0764	0.1308	0.0682	0.083	0.0376
**Girls**						
Intercept	5.26	5.38	5.6	5.76	68.81	66.33
Height (cm)	0.0146	0.0119	0.0128	0.01	0.167	0.1701
Weight (kg)	0.0036	0.0042	0.0051	0.0056	-0.147	-0.150
Age (years)	0.02	0.042	0.0179	0.0376	0.227	0.4539
AIC*	-2,460	-31,192	-2,558	-31,735	-5,648	-37,171
SD (residual)	0.137	0.0766	0.1312	0.0708	0.0831	0.0389

* AIC = Akaike information criterion; Cross-sectional equations from Pérez-Padilla et al. (4) and longitudinal model from reported data. SD = Standard deviation of residuals; FEV_1_ = Forced expiratory volume at 1 s; FVC = Forced vital capacity; FEV_1_/FVC = ratio of FEV_1_ and FVC.

FEV_1_/FVC during growth in the cohort was described adequately only by the cross-sectional Mexican study in boys. Compared with all three equations in girls and with the NHANES III study and the international study for boys, the Z-score of FEV_1_/FVC during longitudinal growth began at zero or below zero and then increased progressively. 

Table 4 compares the multilevel mixed-effects linear equations obtained with our data compared with those of the previous cross-sectional study [4]; both of these fit with the same independent variables. In boys and girls, significant differences were observed for the age co-efficient in all spirometric variables reported. In boys, additional differences were observed for weight co-efficients for FEV_1_ and for FEV_1_/FVC. All of the longitudinal models produced a smaller SD of the residuals and an Akaike information criterion (AIC) [19] lower than that of the cross-sectional models [4]; this indicates that the former were better adjusted, thus preferable to the cross-sectional co-efficients. Spirometric predicted values obtained from multilevel mixed-effects linear models are shown in Table S1 in Online Supporting Information. We obtained the same co-efficients and SD of residuals with the different variance-covariance structures tested.

**Figure 2:**
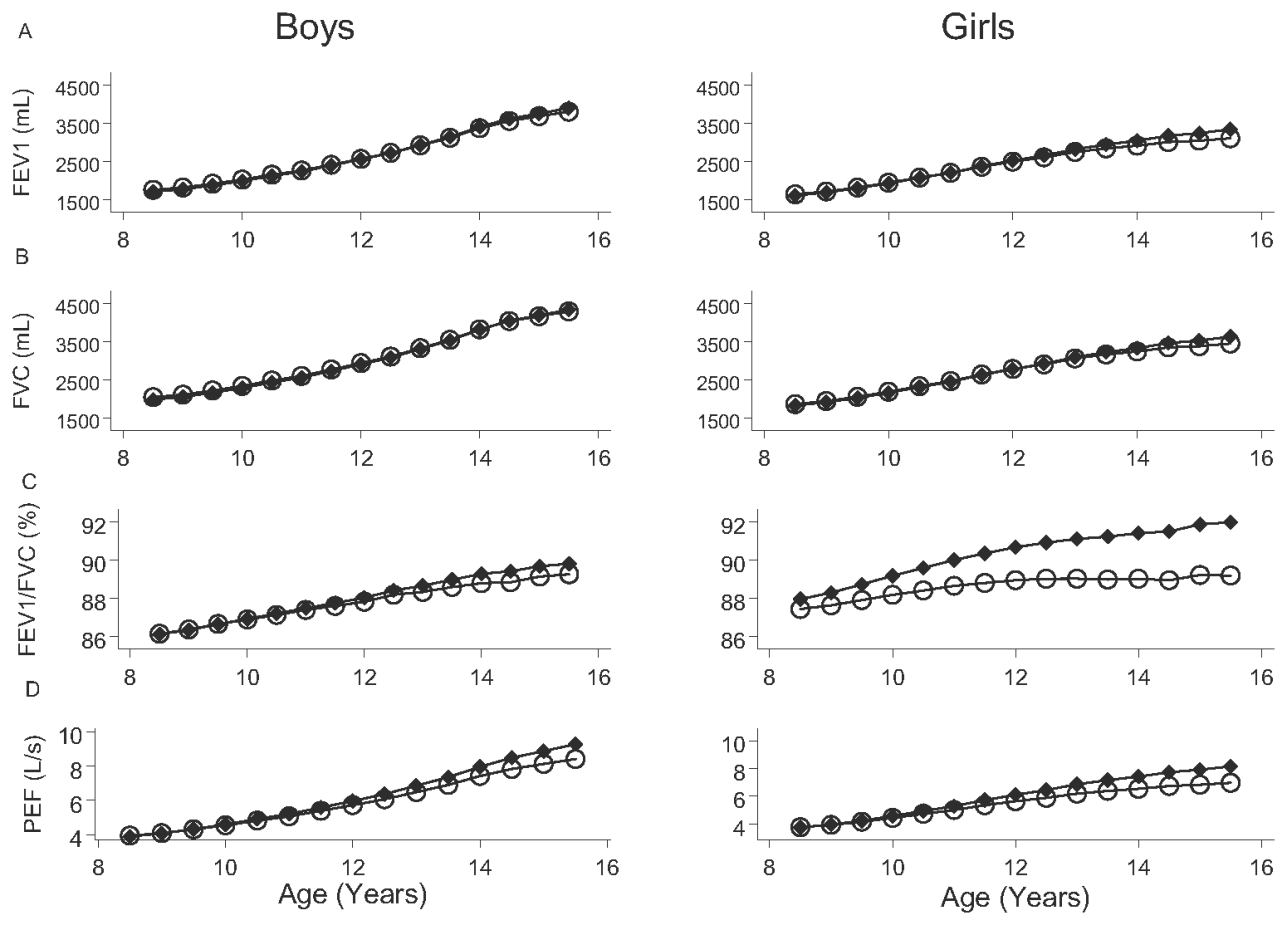
Lung function growth of a cohort of Mexican children as estimated by longitudinal models (full symbols) vs. cross-sectional models (empty symbols). Forced expiratory volume at 1 s (FEV_1_) (panel A), Forced vital capacity (FVC) (panel B), the ratio of FEV_1_ and FVC (FEV_1_/FVC) (panel C) and Peak expiratory flow (PEF) (panel D) vs. age in years of children from Mexico City as expressed in mL (FEV_1_ and FVC) or in percentage (FEV_1_/FVC) for mean age, mean height, and mean weight of children, for longitudinal growth (full circles) or cross-sectional models (empty circles). Estimates from the cross-sectional model and the longitudinal model depart during follow-up for FEV_1_/FVC, as do the values at the end of follow-up, especially for girls.

Figure 2 presents the growth of FEV_1_ (mL), FVC (mL), and FEV_1_/FVC as estimated by the cross-sectional (full symbols) and longitudinal (empty symbols) equations for age and gender, exhibiting mild differences. FEV_1_/FVC during growth in the cohort departs progressively from the cross-sectional equation, especially in girls (Figure 2, panel C).

The percentage of children in the cohort below the Lower limit of normal (LLN, 5^th^ percentile) of the three cross-sectional reference values for FEV_1_, FVC, and their ratio is described in Table S2 in Online Supporting Information. For FEV_1_ and FVC only, the cross-sectional equation for Mexican children generated the expected value of about 5% of measurements <LLN compared with 2.4‒3.3% from the Mexican-American equation [12] and 1.4–2.3% for the international equation [13]. For FEV_1_/FVC, only 3.2‒3.3% were <LLN according to the international equation, with numbers closer to the expected 5% for the Mexican-American equation and to the previous cross-sectional equation for Mexican children [4]. 

**Figure 3:**
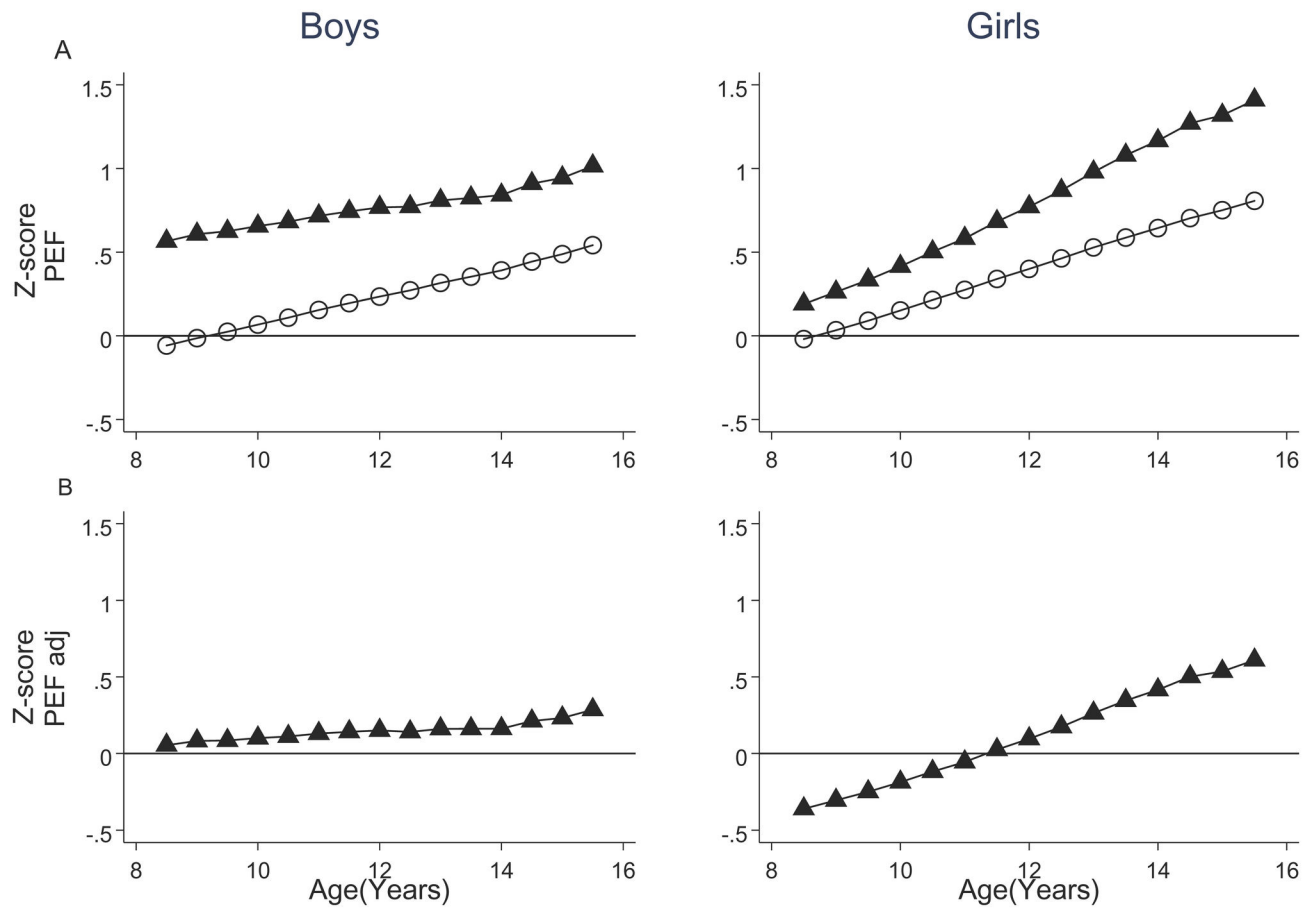
Peak expiratory flow (PEF) growth of a cohort of Mexican children compared with the National Health and Nutrition Examination Survey III (NHANES-III) equation. PEF of children in the cohort expressed as Z-score of the Mexican-American children from the NHANES III study (full triangles, panel A), and Mexican children from a cross-sectional study (empty circles, panel A). Panel B has PEF of Mexican children adjusted to that estimated at sea level. The majority of values for age-, gender- and height-adjusted PEF in Mexican children from the reported cohort are positive compared with those of children among Mexican-American children, and these in addition increase during growth relative to predicted values. Adjusting PEF in Mexico City to sea level values (see Supporting Information) partially reduces the positive bias especially in boys.

Peak expiratory flow (PEF) was also higher in Mexican children compared with Mexican-American children (Figure 3); adjusting PEF values of children from Mexico City to those expected at sea level (see Section C in File S1) reduced, but did not eliminate, differences especially in girls. 

Sitting height was measured in 1,987 Mexican children during the 4^th^ and 5^th^ evaluations (see Table S3). FEV_1_ and FVC remained higher in Mexican children than in Mexican-American children even after adjusting for gender, age, height, weight, sitting height, and their squared terms. In fact, inclusion of sitting height did not significantly increase the explained variability of the models (see Table S4 and Section D in File S1). 

## Discussion

The spirometric function of the studied cohort of Mexican children, adjusted for gender, age, and height, was higher than that described by Quanjer et al. on their compiling spirometric data from children from several countries, including individuals from the U.S. [13]. Compared with the Quanjer equation, mean Z-score for FEV_1_ and FVC was around 0.4, higher than the maximum 0.3 expected for differences in large population samples [14]. The SD was also 10% higher than expected. Spirometric function was also higher than that reported for Mexican-American children from the NHANES III study [12], as described previously [4], showing mean Z-scores for FEV_1_ and FVC values above zero but slightly below 0.3 [14].. However, for these reference values, variation was very close to that expected (SD, 1). Mexican children have a small body compared with Mexican-Americans (see Table S3) and smaller absolute FEV_1_ and FVC, but their lungs are disproportionately large for standing height. Lung function of Mexican children was closer to that of Mexican-Americans compared with standing height. Height has been used in reference equations as a general indicator of body size and lung size [20-22]. Quanjer et al. reported that Mexican children, despite small standing height, did not have a low FEV_1_ and FVC, and also that the FEV_1_/FVC ratio was higher in males than in children from other countries [23]. At the same standing height, Mexican children likely have larger lungs than those in the other studied populations due to a higher sitting height and consequently a longer thorax, or else a wider thorax with the same upper segment height, or both. In the study of Quanjer et al., adjusting spirometric data by sitting height or by sitting height/standing height ratio, reduced the differences among individuals in FEV_1_ and FVC, but not in FEV_1_/FVC [23]. However in our study, adjustment by sitting height in a subsample of Mexican children did not eliminate or reduce differences with Mexican-American children. Because lung function in Mexican children at the same standing height and in a subsample at the same sitting height was higher than in Mexican-American children presumably with a similar genetic background, environmental factors such as nutrition or residence at a high altitude likely explain the differences [24-26].

Mean FEV_1_/FVC departed less from 0 with an SD near 1 and was very similar to that found in Mexican-American children. In our cohort that followed the children from 8–15 years of age, we observed an increase in the FEV_1_/FVC ratio with a plateau in girls (See Tables 1 and 2, and a positive age co-efficient in Table 4), whereas two reference equations predict a decrease in the ratio in the same age range [12,13]. Based on several cross-sectional studies, Quanjer et al. [23] described a differential growth of FEV_1_ and FVC during childhood and adolescence and a complex pattern of their ratio with a decrease from 8 until 10–11 years of age, followed by an increase. 

A decrease in air density with altitude would explain a higher Peak expiratory flow (PEF) and, to a lesser degree, a higher FEV_1_ in Mexico City than at sea level [27], but not the observed increase in FVC nor the age-related changes in FEV_1_, as depicted in Figure 1 compared with reference values (see Supporting Information). However, even taking into account the increase expected with altitude, PEF adjusted by age, gender, and height remained higher in Mexican boys than in Mexican-American children (see Table 3). At present, it is unclear whether a wider thorax, or other factors such as muscle strength or lung compliance [28], better explain the results, but it is clear that collated international reference values from Quanjer et al. [13], and to much lesser extent those deriving from Mexican-American children from the NHANES III study [12], are currently unsuitable for Mexican children residing in Mexico City. The differences found are of such a magnitude as to affect the diagnosis and treatment of children, especially during follow-up. Overall prevalence of functional abnormalities would be underestimated, more with reference values of Quanjer et al. [13] than with those of Hankinson et al. [12]. In addition, if an individual with a chronic disease is tested several times during routine clinical care or during an interventional trial, lung function during growth will appear to change compared with reference values, but spuriously (See Figures 1 and S2 in Online Supporting Information). 

Longitudinal reference equations for Mexican children also differ from the cross-sectional equations reported previously [4], although differences were small and did not change over time except in girls after 12 years of age (see Figure 2). The discrepancies found may well be due primarily to the cohort effect, that is, to the presence of different birth cohorts in the cross-sectional study describing a growth pattern of children that may differ from that observed if the same individuals are followed along time. 

Longitudinal follow-up of the cohort ended at about 15 years of age, during adolescence, but before final height and lung function are reached. In addition, as can be observed in Tables 1 and 2, follow-up decreased during secondary school due to the children’s dropping out of school or to changing to a different school, reducing information at the beginning of adolescence. 

Thus, using the cross-sectional reference values obtained from Mexican children is recommended for this population, at least until a proper longitudinal equation including individuals with final lung growth is available. 

Systematic differences in lung function growth between Mexican children and children from collated international studies [13] are not likely, due to differences in spirometers, spirometric procedures, or technicians, because we employed identical high-quality, volume-based, rolling-seal spirometers and strictly followed a quality-control protocol utilizing international standards [4]. Differences due to sampling error [14] are also unlikely because of the large number of children participating. 

Although one set of reference values with a large sample size due to study design [12] or to the collation of several samples [13] would simplify spirometric testing and interpretation tremendously, it is important to verify whether such reference values are appropriate for the population.

## Conclusions

At the same gender, height, and age, children from Mexico City had higher spirometric lung function than children from several countries reported in an international reference value study and, to a lesser extent, than Mexican-American children. The difference with Mexican-American children did not disappear on adjusting further by sitting height. Whether this is due to a larger lung due to a wider thorax or to other factors is currently unknown. Similarly unknown is whether these changes are due to poor nutrition during childhood or to residence at high altitudes. Cross-sectional reference values published previously [4] currently comprise the best option for Mexican children. 

## Supporting Information

Figure S1 **Longitudinal growth of children’s cohort in weight, Body mass index (BMI), and height.** Means of longitudinal measurements (full squares) compared with measurements obtained from the cross-sectional study (empty circles) (E2). Height was slightly higher in boys from the cross-sectional study, whereas Body mass index (BMI) was higher for the cross-sectional study in both genders. For the cross-sectional reference equation, individuals with BMI >30 were excluded, whereas in the longitudinal study, data from children with BMI >95^th^ percentile according to the Centers for Disease Control and Prevention (CDC) were excluded (to avoid data from children with obesity according to the age-specific definition), resulting in a leaner population.
(TIF)

Figure S2 **Spirometric variables as percentage of predicted values from three reference equations.** Means of longitudinal measurements: Quanjer et al. (empty rhombus sign) (E5), Mexican-American children from the National Health and Nutrition Examination Survey III (NHANES III) (full triangles) (E4), and cross-sectional study from Mexican children (empty circles) (E2). Spirometric values as percentage of three reference studies change over time, yielding a spurious modification of lung function. FEV_1_ = Forced expiratory volume at 1 sec; FVC = Forced vital capacity; FEV_1_/FVC = ratio of FEV_1_ to FVC.
(TIF)

Figure S3 **The Forced expiratory volume at 1 sec (FEV_1_) expected as a function of age according to three reference equations and that found in the cohort in girls (left panel) and boys (right panel).** FEV_1_ found in the cohort (continuous thick line) and that predicted by three cross-sectional reference equations: Quanjer et al. from collated international data (E5) (line with short dashes); Hankinson et al. (line with long dashes) (E4), and a previous study in Mexican children (E2) (continuous thin line). Smoothing performed with LOWESS (Locally weighted smoothing scatterplot).
(TIF)

Figure S4 **Forced vital capacity (FVC) expected as a function of age according to three reference equations and that found in the cohort in girls (left panel) and boys (right panel).** FVC found in the cohort (continuous thick line) and that predicted by three cross-sectional reference equations: Quanjer et al. from collated international data (E5) (line with short dashes); Hankinson et al. (line with long dashes) (E4), and a previous study in Mexican children (E2) (continuous thin line). Smoothing performed with LOWESS (Locally weighted smoothing scatterplot).
(TIF)

File S1 **Methodological Annex.**
(DOCX)

Table S1 **Predicted spirometric values (Standard error, SE) according to three cross-sectional reference values.** Values represent averages of all longitudinal measurements in the cohort (and the Standard error [SE], taking into account study design and repeated measurements with survey commands of the Stata ver. 11.1 software program). For Forced expiratory volume at 1 sec (FEV_1_), the cohort had on average 170 mL higher values than those predicted by Quanjer et al., 70 mL higher than Mexican-Americans from the National Health and Nutrition Examination Survey III (NHANES III) study, and 30 mL higher than those predicted by cross-sectional study. For FVC, similar values were 160, 70, and 10 mL, respectively, and for the FEV_1_ and FVC ratio (FEV_1_/FVC), these were 1.8, 0.4, and 0.5%, respectively. PEF = Peak expiratory flow. L/s = Liter per second; PEFadj = PEF adjusted to values expected at sea level.
(DOCX)

Table S2 **Percentage of children (95% Confidence interval [95% CI]) in the cohort below the lower limit of normal (5^th^ percentile) according to three cross-sectional studies.** Quanjer et al. (E5), Pérez-Padilla et al. (E2), Mexican-Americans from the National Health and Nutrition Examination Survey III (NHANES III) study (E4). From respiratory-healthy children and adequate reference values, 5% of individuals below the Lower limit of normal (LLN) are expected. In addition to overall differences from the expected 5% depicted in the Table, we observed age-related changes (see Figure 1), progressively reducing the prevalence of normal children to the <5^th^ percentile during growth. Underestimation of functional abnormalities is expected, rising with growth toward adolescence. The 95% Confidence intervals [95% CI] took into account survey design and repeated measurements with survey procedures of the Stata ver. 11.1 software program. FEV_1_ = Forced expiratory volume at 1 sec; FVC = Forced vital capacity; FEV_1_/FVC = ratio of FEV_1_ to FVC; PEF = Peak expiratory flow; PEFadj = PEF adjusted to values expected at sea level.
(DOCX)

Table S3 **Main characteristics of children with sitting height measurement by gender.** Mann-Whitney *U* test for differences of medians. *PEF (Peak expiratory flow) adjusted to values expected at sea level, only done in Mexican children. A comparison between the two groups adjusting by several variables is shown in Figure E4.
(DOCX)

Table S4 **Linear regression models for spirometric variables fitted with and without sitting height comparing Mexican children with Mexican-American children.** *P <0.01; **p <0.05; ***p <0.001. For each spirometric variable, two models are shown (columns) as follows: the first with, and the second without sitting height. Inclusion of sitting height did not eliminate differences between populations (site, see Table E3) and improved marginally only the *R* ^2^ of the models.
(DOCX)
