# Supplementary figures and images for: Longitudinal Lung Function Growth of Mexican Children Compared with International Studies
Source: PLoS One. 2013 Oct 15;8(10):e77403. doi: 10.1371/journal.pone.0077403 (PMC3797091; doi:10.1371/journal.pone.0077403)

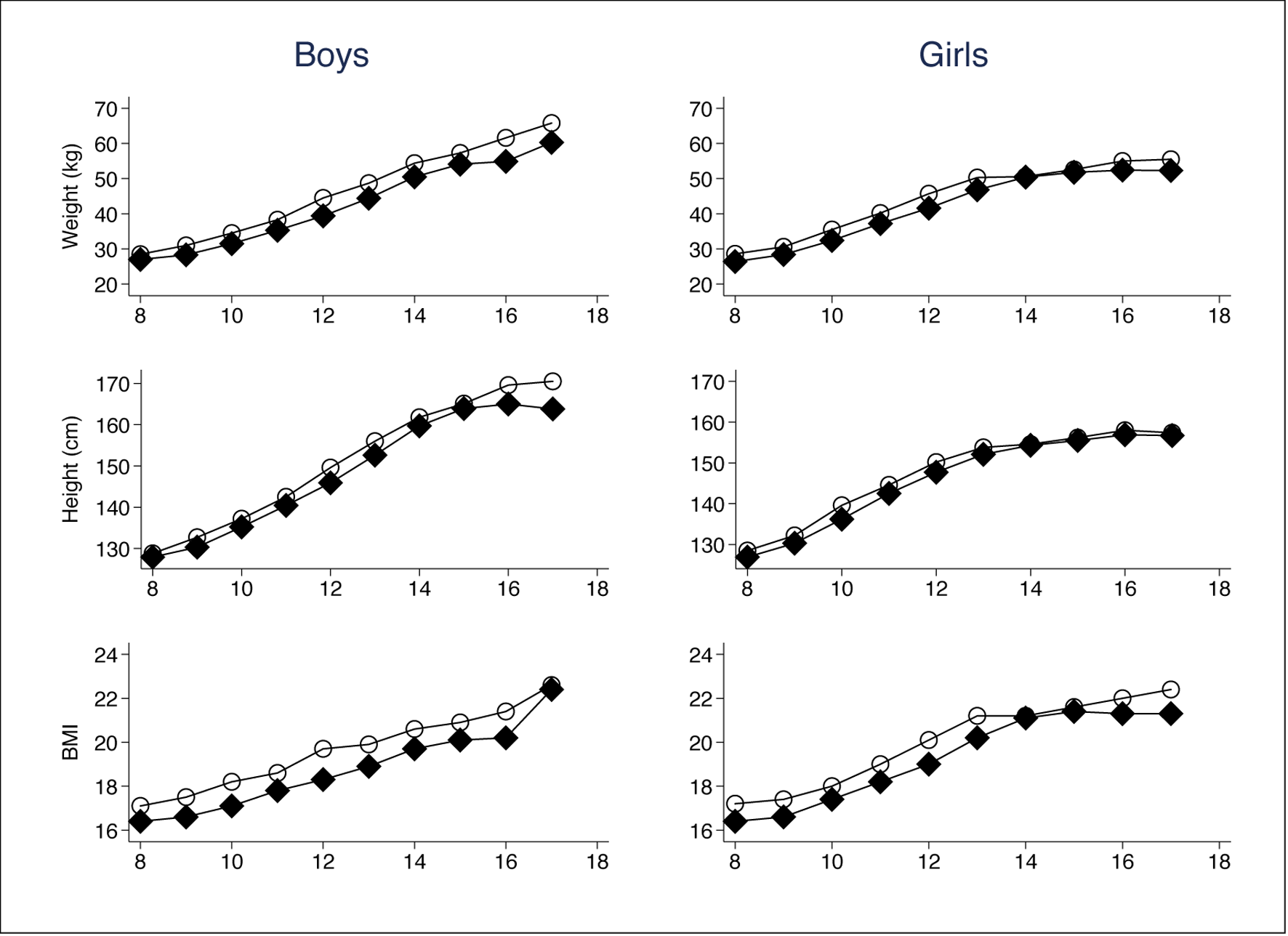

Supplement: Figure S1 — Longitudinal growth of children’s cohort in weight, Body mass index (BMI), and height. Means of longitudinal measurements (full squares) compared with measurements obtained from the cross-sectional study (empty circles) (E2). Height was slightly higher in boys from the cross-sectional study, whereas Body mass index (BMI) was higher for the cross-sectional study in both genders. For the cross-sectional reference equation, individuals with BMI >30 were excluded, whereas in the longitudinal study, data from children with BMI >95th percentile according to the Centers for Disease Control and Prevention (CDC) were excluded (to avoid data from children with obesity according to the age-specific definition), resulting in a leaner population. (TIF) [file pone.0077403.s001.tif]

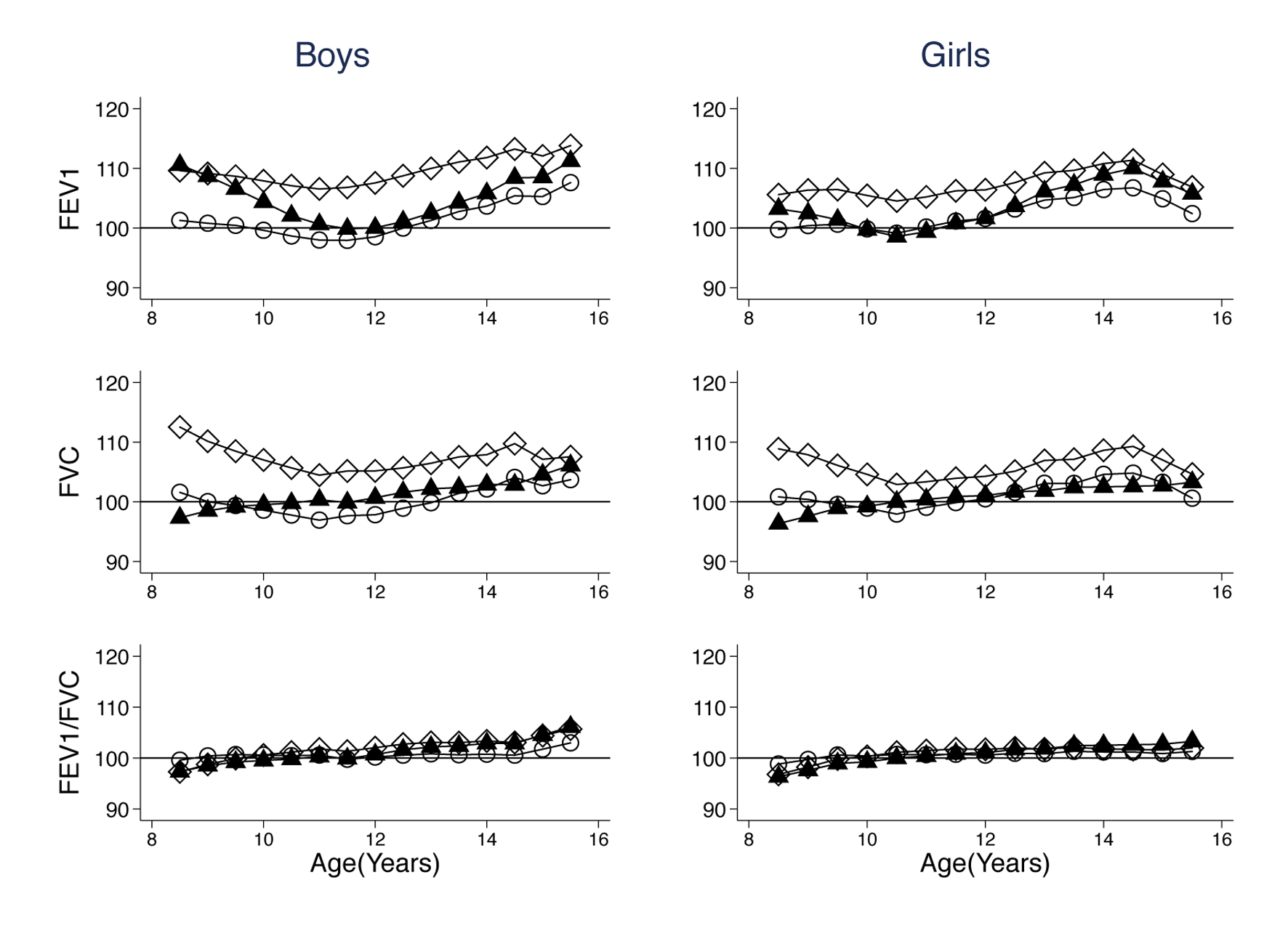

Supplement: Figure S2 — Spirometric variables as percentage of predicted values from three reference equations. Means of longitudinal measurements: Quanjer et al. (empty rhombus sign) (E5), Mexican-American children from the National Health and Nutrition Examination Survey III (NHANES III) (full triangles) (E4), and cross-sectional study from Mexican children (empty circles) (E2). Spirometric values as percentage of three reference studies change over time, yielding a spurious modification of lung function. FEV1 = Forced expiratory volume at 1 sec; FVC = Forced vital capacity; FEV1/FVC = ratio of FEV1 to FVC. (TIF) [file pone.0077403.s002.tif]

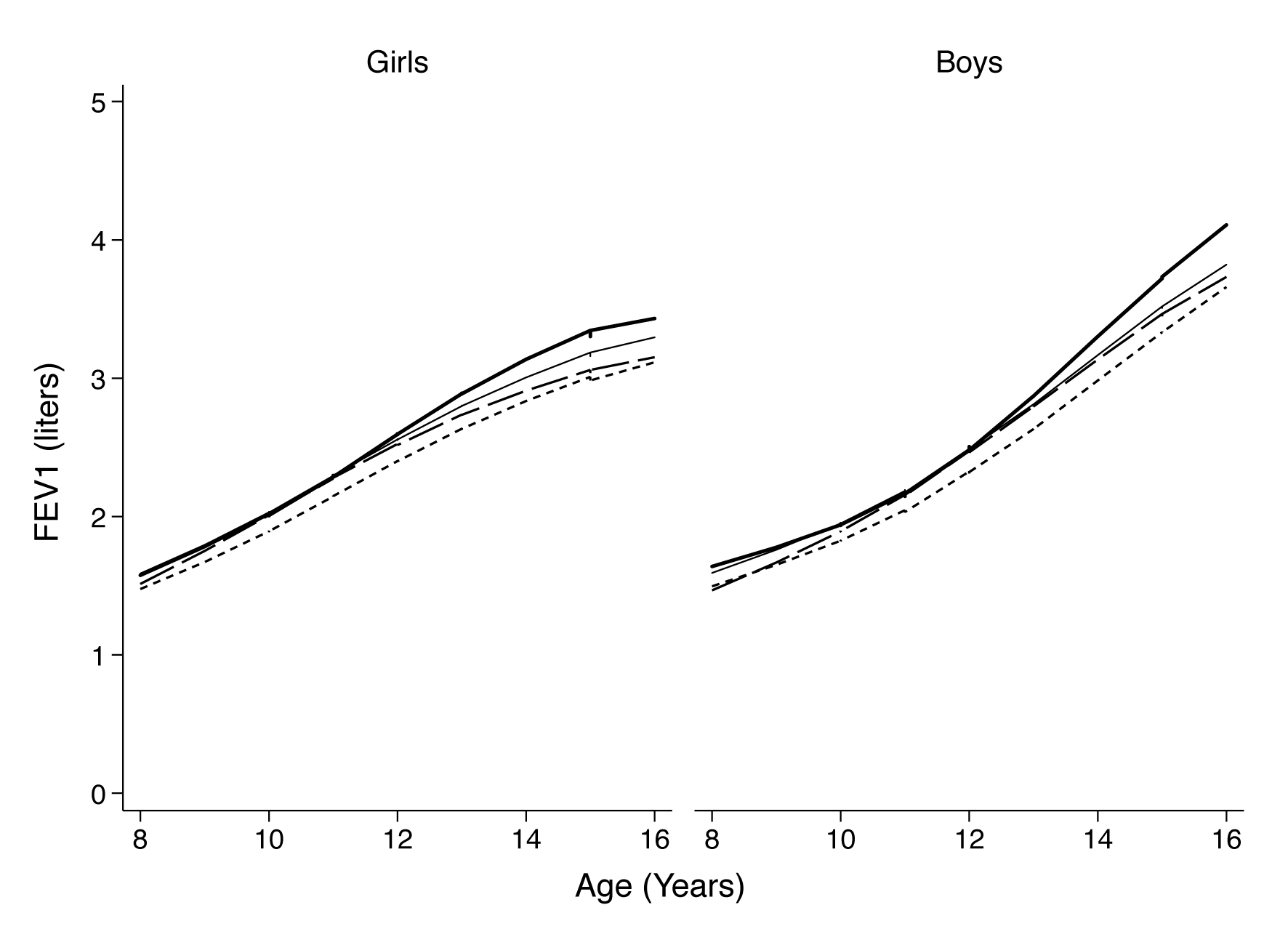

Supplement: Figure S3 — The Forced expiratory volume at 1 sec (FEV1) expected as a function of age according to three reference equations and that found in the cohort in girls (left panel) and boys (right panel). FEV1 found in the cohort (continuous thick line) and that predicted by three cross-sectional reference equations: Quanjer et al. from collated international data (E5) (line with short dashes); Hankinson et al. (line with long dashes) (E4), and a previous study in Mexican children (E2) (continuous thin line). Smoothing performed with LOWESS (Locally weighted smoothing scatterplot). (TIF) [file pone.0077403.s003.tif]

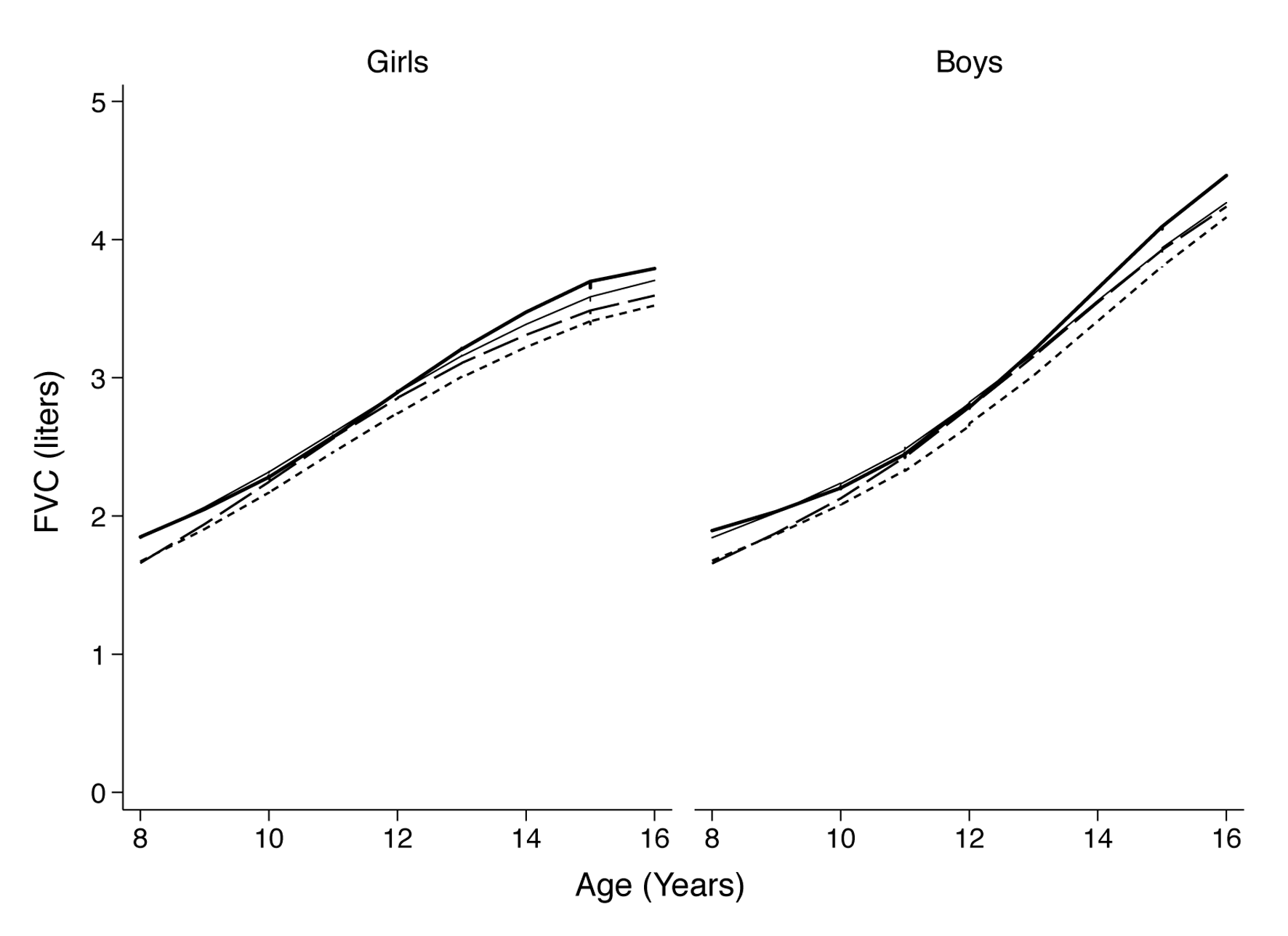

Supplement: Figure S4 — Forced vital capacity (FVC) expected as a function of age according to three reference equations and that found in the cohort in girls (left panel) and boys (right panel). FVC found in the cohort (continuous thick line) and that predicted by three cross-sectional reference equations: Quanjer et al. from collated international data (E5) (line with short dashes); Hankinson et al. (line with long dashes) (E4), and a previous study in Mexican children (E2) (continuous thin line). Smoothing performed with LOWESS (Locally weighted smoothing scatterplot). (TIF) [file pone.0077403.s004.tif]
