# Supplementary material for: Longitudinal Lung Function Growth of Mexican Children Compared with International Studies
Source: PLoS One. 2013 Oct 15;8(10):e77403. doi: 10.1371/journal.pone.0077403 (PMC3797091; doi:10.1371/journal.pone.0077403)
Supplement: File S1 — Methodological Annex. (DOCX) [file pone.0077403.s005.docx]

**Methodological Annex**

***Longitudinal lung function growth of Mexican children compared with international studies***

David Martínez-Briseño, Rosario Fernández-Plata, Laura Gochicoa-Rangel, Luis Torre-Bouscoulet, Rosalba Rojas-Martínez, Laura Mendoza, Cecilia García-Sancho and Rogelio Pérez-Padilla

Short title: Longitudinal lung function growth of Mexican children

**Section A. Study design**

The main objective of the EMPECE (Estudio Metropolitano Para Evaluar los Efectos de la Contaminación en Escolares) study, a prospective study undertaken by the Mexican Ministry of Health in April 1996, was to evaluate the effects of long-term exposure to different levels of ozone (O_3_), particulate matter with a mass median diameter of <10 μm (PM_10_), and Nitrogen dioxide (NO_2_) on lung function growth in children living and attending elementary school in Mexico City. The target population consisted of schoolchildren aged 8 years at baseline, living in Mexico City during the study period and not diagnosed as asthmatic.

Ten fixed-site air-monitoring stations were selected to provide the most complete environmental and meteorological information and to provide a representative sample from all areas of the city. These included one in the Northwest area, two in the Northeast, three in the Central area, two in the Southwest and two in the Southeast. Thirty nine elementary schools within 2 km of the monitoring stations were randomly selected from the elementary school census, which covers both private and public schools, including nine in the Northwest area, 10 in the Northeast, nine in the Central area, six in the Southwest, and five in the Southeast. All third graders in each school were invited to participate in the study. Only children whose parents signed a consent letter were enrolled.

The majority of elementary school students in Mexico City attend school either in the morning (8 am to 2 pm) or in the afternoon (2 pm to 6 pm). Seventy five percent of our study population attended the morning session.

The study population for phase 1 included 1,819 children. This phase consisted of a spirometric test of each child and a baseline questionnaire completed by the child’s parents. A total of 1,351 new participants of the same age (±1 month), as the previously enrolled children were added to the cohort in subsequent phases.

The children were followed every 6 months (spring and fall) for >3 years for up to seven phases. Follow-up consisted of spirometric testing and two questionnaires per phase. One questionnaire, answered at home by the child’s mother, included information on socioeconomic status, the child’s respiratory health history, time spent in different types of activities, outdoor activities, transportation time, Environmental tobacco smoke (ETS) exposure, and history of changes of residence. The second, answered at school by the children and their teachers during school hours, inquired about school time spent in indoor and outdoor activities and ETS exposure.

A number of children were lost to the study, mainly because they moved to another area of the city or to another city altogether. Information was obtained from a total of 3,177 children.

For purposes of the present analysis, children with self-report of asthma, smokers, chronic respiratory symptoms (cough, wheezing, phlegm, dyspnea), or children with >95% percentile of Body mass index (BMI) for age according to growth charts from the Centers for Disease Control and Prevention (CDC) and children <8 years of age were excluded.

**Section B. Statistical models**

Three-level models were fitted to discriminate the sources of variation in the response variables, taking into account the correlation among the repeated measurements. A first level identified the variation between phases within children nested within monitoring stations, a second level identified the variation among subjects within monitoring stations, and a third level identified the variation among monitoring station variables. Y_ijk_ was modeled using multilevel mixed-effects linear regressions by gender. All models were fitted using the maximum likelihood method.

Let the random variable Y_ijk_ denote the FVC, FEV_1_, PEF or FEV_1_/FVC measurement expressed as score Z at phase i for child j in monitoring station k.

Level 1: Between phases (i) within children (j)

Y_ijk_= β_0ij_ + β_1jk_(Height)_ijk_ + β_2jk_(Weight)_ijk_ +β_3jk_(Age)_ijk_ + ε_ijk_

Level 2: Between children within monitoring station (k)

β_0jk_ = γ_00k_ + υ_0jk_

β_1jk_ = γ_10k_ + υ_1jk_

Level 3: Between monitoring stations

γ_00k_ = δ_000_ + δ_001_W_k_ + ν_00k_

γ_10k_ = δ_10k_ + ν_10k_

We computed models with independent, exchangeable, identity, and unstructured variance-covariance matrix form for each dependent variable. We obtained the same co-efficients and Standard deviations (SD) of the residuals with each model.

**Section C. Altitude, air density, and expected maximal expiratory flows**

A decrease in air density proportional to a decrease in barometric pressure occurs with altitude and instantaneous maximal expiratory flows at high lung volumes, such as Peak expiratory flow (PEF), increase with altitude as a function of density^–0.41^ (1). Estimating the increase in PEF due to lower air density in Mexico City, situated at 2,240 m above sea level and with a mean barometric pressure 585 mmHg would be:

PEFmx = PEFsl * (585/760)^–0.41^ = 1.1132679

where PEFmx is the PEF expected in Mexico City based on that measured or predicted at sea level (PEFsl) (E1). This value is consistent with the 14% increase in PEF in children from Mexico City compared with that of Mexican-American children from the National Health and Nutrition Examination Survey III (NHANES III) study (E2 and Table S2). We do not have equivalent predicted values from Stanojevic et al. for PEF.

During acute exposure to altitude at an altitude of 3,457 m (E3), Forced expiratory volume at 1 sec (FEV_1_) increased about 6% and Forced vital capacity (FVC) decreased by a small amount, but the change was not statistically significant. The increase in PEF in Mexico City was nearly that expected for the decrease in air density at an altitude of 2,240 m.

**Section D. Differences in lung function between Mexican children and Mexican-American children adjusting by sitting height**

Sitting height was measured in the 4^th^ and 5^th^ phases of the EMPECE study. We compared the spirometric lung function of Mexican children who had sitting height measurement with that reported in Mexican-American children of the same age from the NHANES III study, using linear regression models to discern whether differences in spirometric lung function between Mexican children and Mexican-American children disappeared or were reduced significantly adjusting by sitting height. At the same standing height, children with shorter legs, and a longer thorax (sitting height), would likely have larger lungs and greater lung function. Shorter legs are commonly associated with undernutrition (E5, E6). Results are shown in Table S4.

REFERENCES

E1. Wood LD, Bryan AC. Effect of increased ambient pressure on flow-volume curve of the lung. *J Appl Physiol* 1969; 27: 4-8.

E2. Pérez-Padilla R, Regalado-Pineda J, Rojas M, Catalán M, Mendoza L, Rojas R, Chapela R, Villalba J, Torres V, Borja-Aburto V, Olaiz G. Spirometric function in children of Mexico City compared to Mexican-American children. *Pediatr Pulmonol* 2003; 35: 177-183.

E3. Gautier H, Peslin R, Grassino A, Milic-Emili J, Hannhart B, Powell E, Miserocchi G, Bonora M, Fischer JT. Mechanical properties of the lungs during acclimatization to altitude. *J Appl Physiol* 1982; 52: 1407-1415.

E4. Hankinson JL, Odencrantz JR, Fedan KB. Spirometric reference values from a sample of the general U.S. population. *Am J Respir Crit Care Med* 1999; 159: 179-187.

E5. Stanojevic S, Wade A, Stocks J, Hankinson J, Coates AL, Pan H, Rosenthal M, Corey M, Lévesque P, Cole TJ. Reference ranges for spirometry across all ages: a new approach. *Am J Respir Crit Care Med* 2008; 177: 253-260.

E6. Malina RM, et al. Secular change in height, sitting height and leg length in rural Oaxaca, southern Mexico: 1968-2000. *Ann Hum Biol* 2004;31(6): 615-633.

E7. Malina RM, Reyes ME, Little BB. Socioeconomic variation in the growth status of urban school children 6-13 years in Oaxaca, Mexico, in 1972 and 2000. *Am J Hum Biol* 2009; 21(6): 805-81
