# Supplementary material for: Longitudinal Lung Function Growth of Mexican Children Compared with International Studies
Source: PLoS One. 2013 Oct 15;8(10):e77403. doi: 10.1371/journal.pone.0077403 (PMC3797091; doi:10.1371/journal.pone.0077403)
Supplement: Table S1 — Predicted spirometric values (Standard error, SE) according to three cross-sectional reference values. Values represent averages of all longitudinal measurements in the cohort (and the Standard error [SE], taking into account study design and repeated measurements with survey commands of the Stata ver. 11.1 software program). For Forced expiratory volume at 1 sec (FEV1), the cohort had on average 170 mL higher values than those predicted by Quanjer et al., 70 mL higher than Mexican-Americans from the National Health and Nutrition Examination Survey III (NHANES III) study, and 30 mL higher than those predicted by cross-sectional study. For FVC, similar values were 160, 70, and 10 mL, respectively, and for the FEV1 and FVC ratio (FEV1/FVC), these were 1.8, 0.4, and 0.5%, respectively. PEF = Peak expiratory flow. L/s = Liter per second; PEFadj = PEF adjusted to values expected at sea level. (DOCX) [file pone.0077403.s006.docx]

Table S1. Predicted spirometric values (Standard error, SE) according to three cross-sectional reference values

|  | Values in the cohort | Pérez- Padilla et al. (E2) | Mexican-Americans (E4) | Quanjer et al. (E5) |
| --- | --- | --- | --- | --- |
| FEV_1_ (L) | 2.39 (0.01) | 2.36 (0.009) | 2.32 (0.008) | 2.22 (0.008) |
| FVC (L) | 2.69 (0.01) | 2.68 (0.01) | 2.62 (0.01) | 2.53 (0.009) |
| FEV_1_/FVC (%) | 89.1 (0.001) | 88.6 (0.03) | 88.7 (0.03) | 87.3 (0.03) |
| PEF (L/s) | 5.69 (0.03) | 5.29 (0.018) | 4.95 (0.016) | - |
| FEV_1_  %P | - | 100.9 (0.22) | 103.0 (0.23) | 107.8 (0.24) |
| FVC %P | - | 99.9 (0.22) | 102.9 (0.24) | 106.1 (0.24) |
| FEV_1_/FVC %P | - | 101.2 (0.12) | 100.4 (0.12) | 101.2 (0.12) |
| PEF (%P) | - | 103.1 (0.18) | 114.7 (0.34) | - |
| PEFadj (%P) | - | - | 101.9 (0.30) | - |

Values represent averages of all longitudinal measurements in the cohort (and the Standard error [SE], taking into account study design and repeated measurements with the survey commands of the Stata ver. 11.1 software program).

For Forced expiratory volume at 1 sec (FEV_1_), the cohort had on average 170 mL higher values than those predicted by Quanjer et al., 70 mL higher than Mexican-Americans from the National Health and Nutrition Examination Survey III (NHANES III) study, and 30 mL higher than those predicted by cross-sectional study. For FVC, similar values were 160, 70, and 10 mL, respectively, and for the FEV_1_ and FVC ratio (FEV_1_/FVC), these were 1.8, 0.4, and 0.5%, respectively. PEF = Peak expiratory flow. L/s = Litre per sec. PEFadj= PEF adjusted to values expected at sea level.
