# Supplementary material for: Longitudinal Lung Function Growth of Mexican Children Compared with International Studies
Source: PLoS One. 2013 Oct 15;8(10):e77403. doi: 10.1371/journal.pone.0077403 (PMC3797091; doi:10.1371/journal.pone.0077403)
Supplement: Table S2 — Percentage of children (95% Confidence interval [95% CI]) in the cohort below the lower limit of normal (5th percentile) according to three cross-sectional studies. Quanjer et al. (E5), Pérez-Padilla et al. (E2), Mexican-Americans from the National Health and Nutrition Examination Survey III (NHANES III) study (E4). From respiratory-healthy children and adequate reference values, 5% of individuals below the Lower limit of normal (LLN) are expected. In addition to overall differences from the expected 5% depicted in the Table, we observed age-related changes (see Figure 1), progressively reducing the prevalence of normal children to the <5th percentile during growth. Underestimation of functional abnormalities is expected, rising with growth toward adolescence. The 95% Confidence intervals [95% CI] took into account survey design and repeated measurements with survey procedures of the Stata ver. 11.1 software program. FEV1 = Forced expiratory volume at 1 sec; FVC = Forced vital capacity; FEV1/FVC = ratio of FEV1 to FVC; PEF = Peak expiratory flow; PEFadj = PEF adjusted to values expected at sea level. (DOCX) [file pone.0077403.s007.docx]

Table S2. Percentage of children (95% Confidence interval [95% CI]) in the cohort below the lower limit of normal (5^th^ percentile) according to three cross-sectional studies

|  | Girls | | | Boys | | |
| --- | --- | --- | --- | --- | --- | --- |
|  | Quanjer et al. (E5) | Pérez- Padilla et al. (E2) | Mexican- Americans (E4) | Quanjer et al. (E5) | Pérez Padilla et al (E2) | Mexican-Americans (E4) |
| FEV_1_ | 2.3 (2.0, 2.7) | 4.1 (3.6, 4.5) | 3.3 (2.9, 3.7) | 1.4 (1.1, 1.7) | 4.9 (4.4, 5.5) | 2.4 (2.0, 2.8) |
| FVC | 2.6 (2.2, 3.0) | 5.0 (4.5, 5.5) | 2.7 (2.4, 3.1) | 2.1 (1.8, 2.5) | 6.5 (5.9, 7.0) | 2.4 (2.0, 2.8) |
| FEV_1_/  FVC | 3.2 (2.8, 3.7) | 3.2 ( 2.8, 3.6) | 5.5 (4.9, 6.0) | 3.3 (2.9, 3.7) | 5.0 (4.5, 5.6) | 6.5 (5.9, 7.1) |
| PEF |  | 2.5 (2.1, 2.8) | 1.3 (1.0, 1.5) |  | 2.7 (2.3, 3.1) | 0.3 (0.2, 0.45) |
| PEFadj |  | - | 2.4 (2.3, 3.2) |  | - | 0.7 (0.5, 0.9) |

Quanjer et al. (E5), Pérez-Padilla et al. (E2), Mexican-Americans from the National Health and Nutrition Examination Survey III (NHANES III) study (E4). From respiratory-healthy children and adequate reference values, 5% of individuals below the Lower limit of normal (LLN) are expected. In addition to overall differences from the expected 5% depicted in the Table, we observed age-related changes (see Figure 1), progressively reducing the prevalence of normal children to < 5^th^ percentile during growth. Underestimation of functional abnormalities is expected, rising with growth toward adolescence. The 95% Confidence intervals [95% CI] took into account survey design and repeated measurements with the survey procedures of the Stata ver. 11.1 software program. FEV_1_ = Forced expiratory volume at 1 sec; FVC = Forced vital capacity; FEV_1_/FVC = ratio of FEV_1_ to FVC; PEF=Peak expiratory flow, PEFadj=PEF adjusted to values expected at sea level
