# Supplementary material for: Longitudinal Lung Function Growth of Mexican Children Compared with International Studies
Source: PLoS One. 2013 Oct 15;8(10):e77403. doi: 10.1371/journal.pone.0077403 (PMC3797091; doi:10.1371/journal.pone.0077403)
Supplement: Table S3 — Main characteristics of children with sitting height measurement by gender. Mann-Whitney U test for differences of medians. *PEF (Peak expiratory flow) adjusted to values expected at sea level, only done in Mexican children. A comparison between the two groups adjusting by several variables is shown in Figure E4. (DOCX) [file pone.0077403.s008.docx]

Table S3. Main characteristics of children with seating height measurement by gender

| Variable | HNANES III | EMPECE | P value |
| --- | --- | --- | --- |
| **Boys** | (n=510) | (n=938) |  |
| Age (years) | 11.9 (3.8) | 10.5 (0.9) | P <0.001 |
| Height (cm) | 147.1 (25.6) | 138.0 (9.0) | P <0.001 |
| Sitting height (cm) | 75.4 (12.2) | 72.0 (5.9) | P < 0.001 |
| Weight (kg) | 42.3 (23.8) | 33.0 (9.0) | P < 0.001 |
| BMI (kg/m2) | 18.8 (5.0) | 17.2 (3.3) | P < 0.001 |
| FEV_1_ (L) | 2.46 (1.34) | 2.12 (0.42) | P < 0.001 |
| FVC (L) | 2.88 (1.49) | 2.42 (0.52) | P < 0.001 |
| FEV_1_/FVC (%) | 87.7 (7.2) | 88.6 (7.3) | 0.039 |
| PEF (L/s) | 5.2 (2.8) | 5.1 (1.2) | 0.246 |
| PEFadj (L/s)* | 5.2 (2.8) | 4.6 (1.1) | P < 0.001 |
|  |  |  |  |
| **Girls** | (n=503) | (n=1049) |  |
| Age (years) | 12.2 (4.1) | 10.4 (0.7) | P < 0.001 |
| Height (cm) | 150.2 (19.6) | 139.0 (10.0) | P < 0.001 |
| Sitting height (cm) | 78.5 (11.8) | 73.0 (6.4) | P < 0.001 |
| Weight (kg) | 45.3 (21.0) | 34.0 (10.0) | P < 0.001 |
| BMI (kg/m^2^) | 20.1 (5.8) | 17.5 (3.9) | P < 0.001 |
| FEV_1_ (L) | 2.48 (1.12) | 2.05 (0.48) | P < 0.001 |
| FVC (L) | 2.77 (1.26) | 2.29 (0.51) | P < 0.001 |
| FEV_1_/FVC (%) | 90.3 (6.8) | 90.4 (0.5) | 0.509 |
| PEF (L/s) | 5.3 (2.2) | 5.1 (1.3) | 0.058 |
| PEFadj (L/s)* | 5.3 (2.2) | 4.6 (1.2) | P < 0.001 |

Mann-Whitney U test for differences of medians

* PEF adjusted to values expected at sea level, only done in Mexican children. A comparison between the two groups adjusting by several variables is shown in figure E4.
