# Supplementary material for: Longitudinal Lung Function Growth of Mexican Children Compared with International Studies
Source: PLoS One. 2013 Oct 15;8(10):e77403. doi: 10.1371/journal.pone.0077403 (PMC3797091; doi:10.1371/journal.pone.0077403)
Supplement: Table S4 — Linear regression models for spirometric variables fitted with and without sitting height comparing Mexican children with Mexican-American children. *P <0.01; **p <0.05; ***p <0.001. For each spirometric variable, two models are shown (columns) as follows: the first with, and the second without sitting height. Inclusion of sitting height did not eliminate differences between populations (site, see Table E3) and improved marginally only the R 2 of the models. (DOCX) [file pone.0077403.s009.docx]

Table S4. Linear regression models for spirometric variables fitted with and without sitting height comparing Mexican children with Mexican-American children (site)

| Variable | Log(FEV_1_) | |  | Log(FVC) | |  | FEV_1_/FVC | |
| --- | --- | --- | --- | --- | --- | --- | --- | --- |
|  | Coefficient | Coefficient |  | Coefficient | Coefficient |  | Coefficient | Coefficient |
| **Boys** |  |  |  |  |  |  |  |  |
| Site | 0.0356* | 0.0435*** |  | 0.0324*** | 0.0389*** |  | 0.3075 | 0.4320 |
| Height (cm) | 0.0238* | 0.0057 |  | 0.0325** | 0.0166* |  | -0.7579** | -0.9551*** |
| Sitting height (cm) | -0.0554** |  |  | -0.0485** |  |  | -0.6194 |  |
| Weight (kg) | 0.0043** | 0.0035** |  | 0.0068*** | 0.0061*** |  | -0.2198** | -0.2285** |
| Age (years) | 0.0088 | -0.0118 |  | 0.0240 | 0.0063 |  | -1.3529 | -1.6090 |
| Height^2^ | -0.00004 | 0.00003 |  | -0.0001 | -0.00001 |  | 0.0028** | 0.0036*** |
| Sitting height^2^ | 0.0004** |  |  | 0.0004** |  |  | 0.0048 |  |
| Weight^2^ | -0.000032** | -0.000019 |  | -0.00004** | -0.00003** |  | 0.0007 | 0.0009 |
| Age^2^ | 0.0005 | 0.0015 |  | -0.0001 | 0.0007 |  | 0.0569 | 0.0692 |
| Constant | -0.1795 | -0.7890 |  | -1.0273** | -1.5606** |  | 173.8*** | 166.9*** |
| R^2^ | 0.776 | 0.771 |  | 0.782 | 0.779 |  | 0.053 | 0.051 |
| AIC | -2100.798 | -2074.858 |  | -2111.328 | -2099.168 |  | 8923.263 | 8943.155 |
|  |  |  |  |  |  |  |  |  |
| **Girls** |  |  |  |  |  |  |  |  |
| Site | 0.0415* | 0.0423*** |  | 0.0443*** | 0.045*** |  | -0.2763 | -0.2607 |
| Height (cm) | -0.0016 | 0.0277* |  | 0.0040 | 0.0279** |  | -0.4831 | -0.0099 |
| Sitting height (cm) | 0.0611* |  |  | 0.0493* |  |  | 1.0360 |  |
| Weight (kg) | 0.0059* | 0.0073*** |  | 0.0087* | 0.0098*** |  | -0.256*** | -0.231** |
| Age (Year) | 0.0255 | 0.0424 |  | 0.0222 | 0.036 |  | 0.2356 | 0.5562 |
| Height^2^ | 0.00005 | -0.0001 |  | 0.0000 | 0.000 |  | 0.0021 | 0.0004 |
| Sitting height^2^ | -0.0004* |  |  | -0.0003* |  |  | -0.0067 |  |
| Weight^2^ | -0.00003* | -0.00004** |  | -0.00004** | -0.000048** |  | 0.0012* | 0.0010 |
| Age^2^ | -0.000012 | -0.0006 |  | 0.0001 | -0.00039 |  | -0.0079 | -0.0200 |
| Constant | -2.86* | -2.77** |  | -2.69*** | -2.62** |  | 83.61** | 86.36** |
|  |  |  |  |  |  |  |  |  |
| R^2^ | 0.731 | 0.728 |  | 0.737 | 0.736 |  | 0.034 | 0.033 |
| AIC | -2162.812 | -2145.617 |  | -2179.527 | -2177.600 |  | 9409.467 | 9469.116 |

* P < 0.01 ** P < 0.05 *** P < 0.001

For each spirometric variable two models are shown (columns), the first with and the second without sitting height. Inclusion of sitting height did not eliminate differences between populations (site, see Table E3) and improved only marginally the R^2^ of the models.
